# Genomic Analysis of Taurine and Indicine Ancestry in the Montana Tropical Composite Population

**DOI:** 10.1111/jbg.70017

**Published:** 2025-09-29

**Authors:** Camila Alves dos Santos, El Hamidi Hay, Elisangela Chicaroni de Mattos Oliveira, Rafael Espigolan, José Bento Sterman Ferraz, Tiago do Prado Paim

**Affiliations:** ^1^ Programa de Pós‐graduação Em Zootecnia Instituto Federal de Ciência, Educação e Tecnologia Goiano Rio Verde Goiás Brazil; ^2^ Department of Animal Sciences Purdue University West Lafayette Indiana USA; ^3^ USDA Agricultural Research Service, Fort Keogh Livestock and Range Research Laboratory Miles City Montana USA; ^4^ Departamento de Zootecnia, Faculdade de Zootecnia e Engenharia de Alimentos Universidade de São Paulo São Paulo Brazil

**Keywords:** beef cattle, biological type, complementarity, crossbreeding

## Abstract

The Montana Tropical cattle, a Taurine and Indicine composite, were developed in Brazil since 1994 and were based on crossing four biological types of cattle: zebu (mainly Nelore), tropical adapted taurine (mainly Senepol and Romosinuano), British taurine (mainly Angus) and continental taurine (as Charolais, Simental and Limousin). This study aimed to characterise the genetic ancestry of this composite breed at the genomic level. Principal component analysis revealed the composite in intermediate space between indicine and taurine but closer to the Taurine cluster, which is consistent with its multi‐breed origin. The ADMIXTURE analysis indicated the Montana Tropical to be composed of several progenitor breeds without an indication of a dominant breed. Local ancestry analysis showed the Montana animals to have an average of 24% (standard deviation of ±5.41) Zebu ancestry. The total taurine ancestry was 62%, consisting of 19.6% (±6.96) from tropical adapted taurine, 20.1% (±5.99) from British taurine, 21.92% (±7.79) from continental taurine and 14% (±5.26) of the genome was undetermined. Based on the pedigree, these animals would have 20.8% ± 8.5% Zebu, 50.5% ± 14.4% tropical adapted taurine, 21.1% ± 13.5% British taurine and 7.6% ± 5.1% of continental European taurine in their composition. The genomic regions in the composite originating from each biological type highlight the trait complementarity each genetic group contributes. For example, the genomic region of high tropical adapted taurine ancestry was shown to harbour the slick hair locus, and the regions of high indicine ancestry are associated with high length of productive life. This study unravels the complex genetic ancestry of the Montana Tropical composite, highlighting the effective blend of ancestral gene pools that enhance key production and adaptation traits.

## 1 Introduction

Composites are an excellent tool to combine favourable traits from various breeds. The composite populations are formed by crossing individuals from two or more breeds with the purpose of exploiting heterosis and complementarity between them (Gregory et al. 1992, 1994). The heterosis component in composite populations has shown to be of economic benefit in agriculture (Lippman and Zamir 2007). For example, heterosis was reported to increase milk yield by approximately 15%, calf weaning weight by 10% to 20%, and cow lifetime productivity by 25% to 30% (Gregory et al. 1992, 1994).

Recently, several efforts have been made to understand the genetic architecture and the ancestry of composite cattle breeds, aiming to clarify how breed complementarity and selection work on a genomic level (Paim et al. 2022). Focus has been on indicine × taurine composites, because it is easier to differentiate the ancestry of these subspecies. These previous studies always identified lower indicine genomic contributions than expected based on the pedigree (Paim et al. 2020; Crum et al. 2021; Mulim et al. 2022; Vahedi et al. 2022). These papers demonstrated how complementarity and selection jointly contribute to shaping the genetic architecture of the Composite breeds population.

Mulim et al. (2022) studied Purunã breed, a four‐breed composite population, and demonstrated a low consistency of gametic phase with the founder breeds. It seems that composite breeds formed by more than two breeds and with multiple taurine and indicine founders may have a different genetic architecture than previously studied two‐breed composite populations (Grigoletto et al. 2020).

The Montana Tropical composite was developed in Brazil with the objective of meeting the national needs of higher meat quality produced in extensive systems and in tropical climate conditions (Ferraz et al. 1999). This composite population was formed by crossbreeding several breeds based on four biological groups, named as: N: indicine breeds; A: tropical adapted taurine; B: British taurine and C: continental European taurine. The initial contribution of each group based on pedigree varied from N (0% to 37.5%), A (12.5% to 87.5%), B (0% to 75%) and C (0% to 75%). For registration in the herd book association, animals must have at least three groups' contributions on the pedigree.

The Montana breed combination was planned based on MARC II composites (Gregory et al. 1999) of the U.S. Meat Animal Research Center. The idea was to obtain males capable of natural mating with Nelore females in tropical conditions, maintaining some level of heterosis. Simultaneously, good carcass and meat quality traits were sought while maintaining the robustness and adaptation of these animals to harsh tropical environments (Hansen 2004; Santana Jr et al. 2013; Xia et al. 2023; Santos et al. 2024). After initial crossbreeding, this population has undergone artificial selection focused on weaning weight, yearling weight, muscularity and reproduction (scrotal circumference or probability of precocious pregnancy). Therefore, in this study, we aim to elucidate how the highly diverse genomic composition due to initial crossbreeding contributes to the actual genomic architecture after almost 30 years of selection in a tropical environment. This study utilised genomic information from several breeds to understand the ancestry in the Montana composite population and characterise the conserved founder breeds' genomic regions.

## 2 Material and Methods

### 2.1 Genotypes

The genotypic data consisted of the founder biological types and the Montana Tropical (Table 1). The founders' genotypes were obtained from WIDDE (http://widde.toulouse.inra.fr/), a public repository; NAGP (National Animal Germplasm Program, Agricultural Genetic Resources Preservation Research: Fort Collins, CO); and GMAB (Animal Improvement and Biotechnology Group of the Faculty of Animal Science and Food Engineering: Pirassununga, São Paulo, Brazil). All the founders were genotyped with the High‐density SNP panel (777,962 SNP, BovineHD Beadchip, Illumina, San Diego, CA, United States) (Table 1). The genotypes of Montana Tropical varied in SNP density and were imputed to a common 54,791 SNPs. For the imputation process, 1342 animals were used as the reference population. The target population consisted of 1893 animals, of which 503 were genotyped with the GGP LD BeadChip 35 K (35,237 SNPs; GGP indicus; Neogen Company, Lansing, MI, USA) and 1390 with the GGP LD BeadChip 30 K (GGP indicus; Neogen Company, Lansing, MI, USA). Imputation was performed using FImpute version 3.0. The resulting genotypic data consisted of 3215 animals with 50 K SNP (51,692 SNPs) which were used for the local ancestry study.

**TABLE 1:** Summary of the founder breed groups of the Montana Tropical composite.

Biological type	BT^a^	Population	Pop^a^	Source	Samples
Composite	MTN	Montana Tropical	MTN	GMAB	3201
					16
Indicine	IND	Nelore	NEL	GMAB	2444
				WIDDE	31
		Brahman	BRM	WIDDE	46
				NAGP	67
Tropical adapted taurine	ADP	Senepol	SEN	WIDDE	12
				NAGP	24
		Romosinuano	ROM	NAGP	20
		Tuli	TUL	NAGP	12
British taurine	BRT	Angus	ANG	NAGP	95
				WIDDE	52
		Hereford	HFD	WIDDE	35
Continental European taurine	CNT	Simental	SIM	WIDDE	10
		Limousin	LMS	WIDDE	50
		Charolais	CHL	NAGP	67
Outgroup	OUTG	*Bubalus depressicornis*	OWB	WIDDE	6

^a^ Acronyms of the different biological types and populations.

The founders and Montana Tropical data were merged using the ‐‐merge function from the software PLINK 1.9 (Chang et al. 2015) with a total of 41,099 common SNPs. For the quality control of the merged data, minor allele frequency control (‐‐maf > 1%) was applied, removing a total of 453 variants, leaving 40,646 variants in the dataset for the local ancestry analyses on 29 autosomal chromosomes.

### 2.2 PCA and ADMIXTURE

For principal component and ADMIXTURE analyses, the genotypes were pruned considering a window size of 50 SNPs with a step size equal to 5 and r^2^ threshold higher than 0.5 (‐‐indep‐pairwise 50 5 0.5), and 32,871 SNPs were kept. As the large imbalance between the number of animals of each breed can bias ADMIXTURE results, we ran these analyses on a reduced dataset with 796 genotypes. To get this number of genotypes, we randomly selected 150 Nelore samples and 150 Montana samples, always aiming to retain the representation of the diversity inside the breed. PLINK 1.9 (Chang et al. 2015) was used to perform Principal Component Analysis (PCA) aiming to visually verify the integrity of the data after the merging step. PCA was performed with up to 30 principal components, retaining only components with eigenvalues greater than 1, following Kaiser's rule (Kaiser 1991). The individual eigenvectors were plotted along the different axes of a graph. The total variance explained by the main principal components was also estimated. ADMIXTURE analysis was performed using the unsupervised maximum likelihood method implemented in the ADMIXTURE program version 1.3.0. The *K* values (parameter that describes the number of subpopulations that make up the total population in a dataset provided as input) of 2, 4 and 16 were used to establish the clustering patterns in the population (Alexander et al. 2009). To determine the best *K* value for the population, *K* from 2 to 20 was tested. We identified the optimum *K* based on CV error (Figure S1). All the plots were generated using the R package pophelper 2.3.1 (Francis 2017).

### 2.3 Local Ancestry

For the estimation of ancestry of the different biological types in the Montana Tropical population, we performed a supervised method of ghap v2.1 (Utsunomiya et al. 2020). We followed the steps recommended by Utsunomiya et al. (2020), as follows: (i) construction of prototype alleles (i.e., allele frequencies) for each biological type (Indicine, tropical‐adapted taurine, British taurine and Continental European taurine); and (ii) segmental classification of test haplotypes (Montana Tropical) according to the generated prototype alleles using sliding windows and Euclidean distances.

With the assigned haplotypes of the four distinct lineages, we calculated the proportion of haplotypes attributed to each lineage at the individual SNP location of the Montana Tropical population. This provided a percentage‐based representation of the sample's ancestral composition for every SNP site examined. The results were separated into four Manhattan plots, one for each biological type, where we examined the genomic regions with high ancestry for each biological type based on the top 1% considering a gaussian distribution. We used this threshold to capture only genomic regions with very strong deviation from the expected based on the whole genome ancestral composition.

### 2.4 Gene and QTL Annotation

For the gene and Quantitative Trait Loci (QTL) annotation, we used the top 1% regions of ancestry proportion for each biological type as the threshold. The GALLO package (Fonseca et al. 2020) was used with data for *Bos taurus* from the Ensembl database (www.ensembl.org/Bos_taurus/Info/Index, version ARS‐UCD1.2) for gene annotation, enrichment analyses and figure generation.

## 3 Results

**FIGURE 1:**
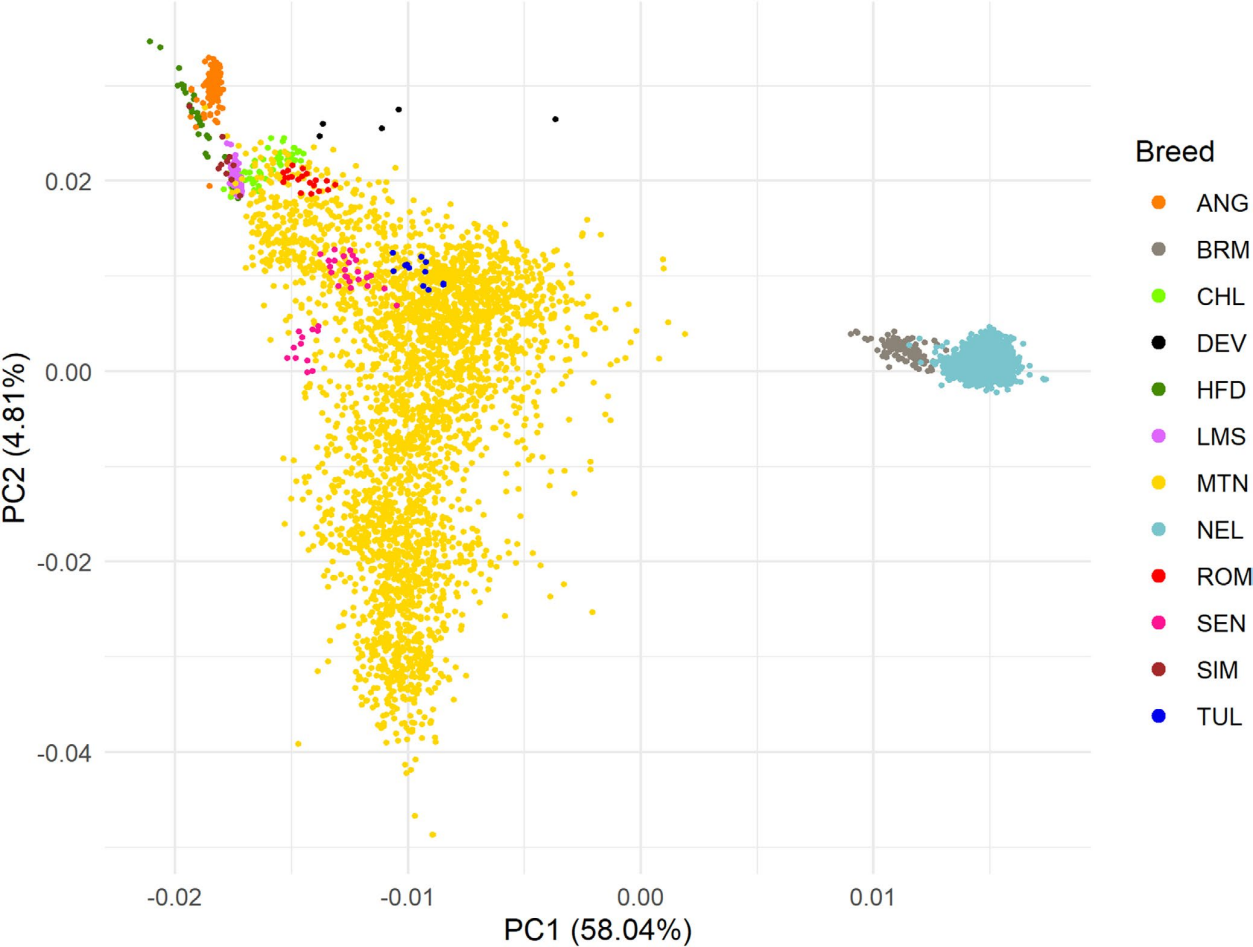
First two components of principal component analysis using genomic data of Montana Tropical animals and founders. ANG, Angus; BRM, Brahman; CHL, Charolais; HFD, Hereford; LMS, Limousin; MTN, Montana Tropical; NEL, Nelore; OWB, *Bubalus depressicornis* ; ROM, Romosinuano; SEN, Senepol; SIM, Simental; TUL, Tuli. [Colour figure can be viewed at wileyonlinelibrary.com]

The principal component analysis showed the first two principal components to capture the population structure among the breeds (Figure 1). The proportion of variance explained was 53.90% for PC1 and 5.32% for PC2. Principal component 1 captured variation seemingly related to the divergency of *Bos taurus* versus *Bos indicus* , with the Montana Tropical composite being intermediate. Additionally, the Montana Tropical composite cluster shows higher variation. As expected, African Tuli and Senepol (tropical adapted taurine) are clustering with the Montana Tropical population. The second principal component (PC2) primarily captured variation within *Bos taurus* breeds, separating the Hereford and Angus and other taurine breeds.

**FIGURE 2:**
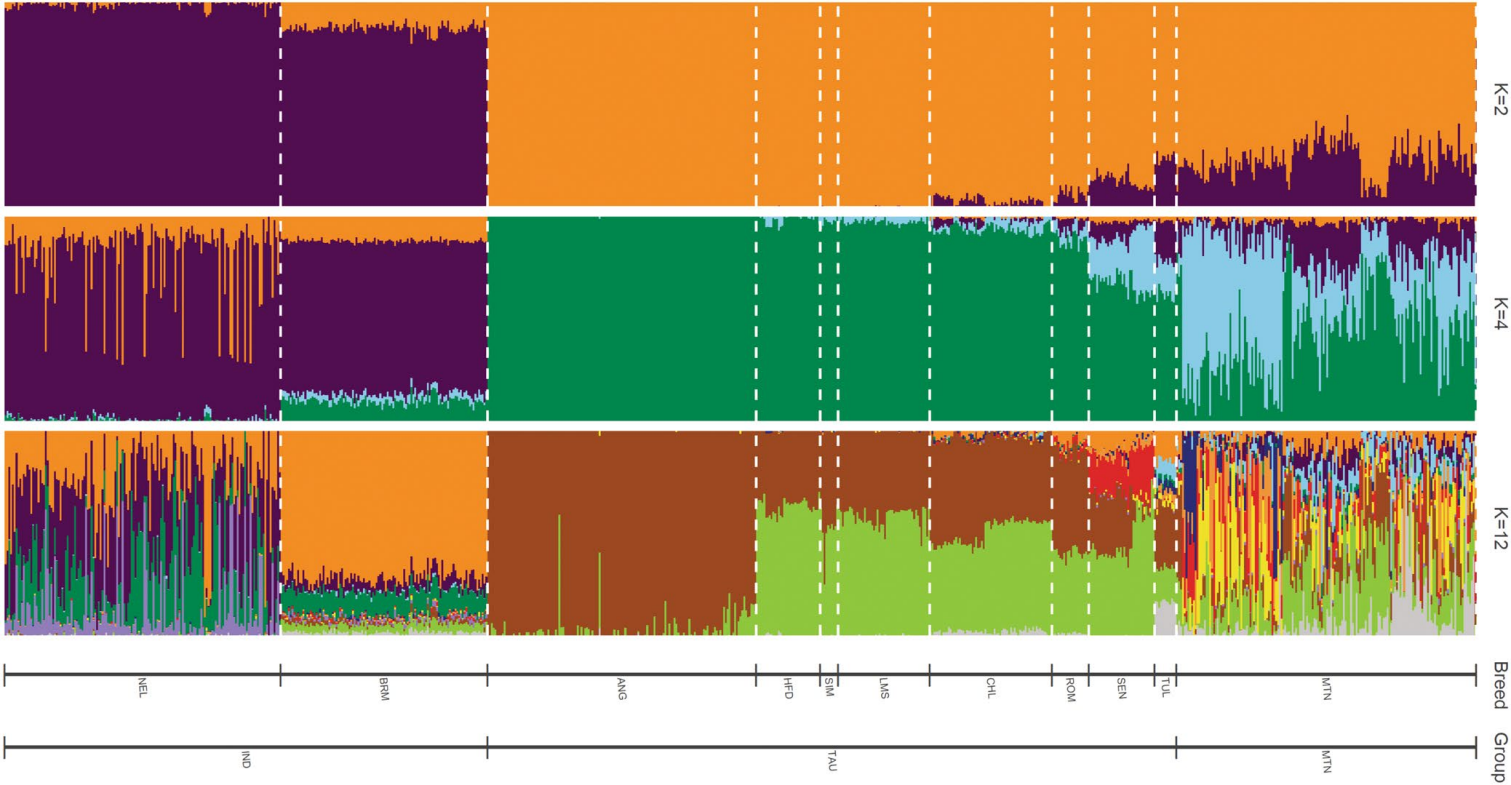
Population structure of composite Montana Tropical and founders populations inferred by ADMIXTURE software. Each animal is represented by a single vertical bar divided into *K* colours, where *K* is the number of assumed ancestral groupings, which is plotted from *K* equal to 2, 4 and 16. ANG, Angus; BRM, Brahman; CHL, Charolais; HFD, Hereford; IND, Indicine; LMS, Limousin; MTN, Montana Tropical; NEL, Nelore; OUTG, Outgroup; OWB, Bubalus *depressicornis* ; ROM, Romosinuano; SEN, Senepol; SIM, Simental; TUL, Tuli; TAU, Taurine. [Colour figure can be viewed at wileyonlinelibrary.com]

The unsupervised ADMIXTURE estimates of all animals are shown in Figure 2. Montana Tropical is represented by multiple ancestry groups as expected. Additionally, no apparent specific breed was overrepresented. At *K* = 2 and 4, we can see the Montana Tropical represented by more Taurine than Indicine contribution.

### 3.1 Local Ancestry

The Montana animals had an average of 24% (standard deviation of ±5.41) Zebu ancestry. The total taurine ancestry was 62%, consisting of 19.6% (±6.96) from tropical adapted taurine, 20.1% (±5.99) from British taurine, and 21.92% (±7.79) from Continental European taurine. The method was unable to determine the ancestry of 14% (±5.26) of the genome. Based on the pedigree data, these animals would have 20.8% ± 8.5% Zebu, 50.5% ± 14.3% tropical adapted taurine, 21.1% ± 13.5% British taurine and 7.6% ± 5.1% of Continental European taurine in their composition. Therefore, it seems that the local ancestry method mainly underestimated the tropical adapted taurine counterpart.

**FIGURE 3:**
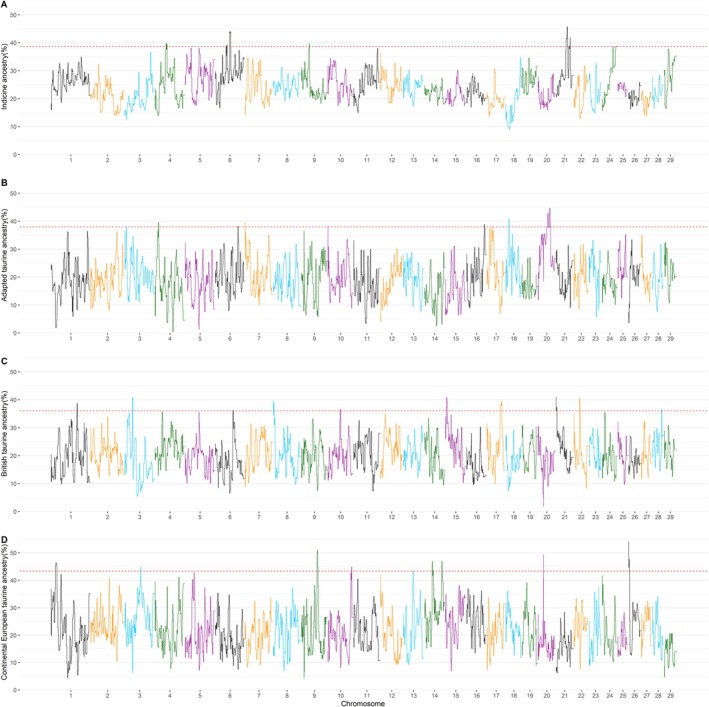
Manhattan plot of local ancestry. (A) Indicine, (B) Adapted taurine, (C) British taurine, (D) Continental European taurine across 3217 Montana Tropical animals. [Colour figure can be viewed at wileyonlinelibrary.com]

Figure 3 shows Manhattan plots of the ancestry of each region in the genome of the Montana Tropical animals. Regions along the genome differed in their ancestry; no chromosome showed a predominant ancestry of a particular biological type. The top 1% of genomic regions of ancestry from the four biological types are presented in Table 2. Notably, autosome 6 stood out with admixed regions containing Indicine, tropical‐adapted taurine and British taurine ancestry.

**TABLE 2:** The top 1% of genomic regions of founders' introgression in the Montana Tropical animals.

Biological type	Chr	Start (BP)	End (BP)	Size	Number of SNP's	Number of genes	Biological type (%) Min–Max
Indicine	4	43,401,613	49,879,377	6,477,764	42	78	39.35–39.35
	6	45,679,748	48,940,656	3,260,908	21	9	39.30–39.30
	6	57,059,899	62,688,209	5,628,310	63	78	43.93–49.93
	9	30,907,526	34,150,181	3,242,655	21	41	39.66–39.66
	21	39,530,170	48,851,905	9,321,735	105	95	38.87–45.72
	21	55,833,392	58,765,781	2,932,389	63	45	39.60–41.98
	24	54,897,524	62,640,230	7,742,706	116	119	38.77–38.77
Adapted taurine	3	5,731,076	8,379,108	2,648,032	21	48	38.01–38.01
	4	12,817,101	15,988,405	3,171,304	21	40	39.57–39.57
	6	89,863,255	92,958,509	3,095,254	21	46	38.24–38.24
	7	1	1,715,134	1,715,133	21	36	39.60–39.60
	10	1	2,223,455	2,223,454	21	23	38.20–38.20
	16	70,855,127	74,048,396	3,193,269	21	61	38.84–38.84
	17	19,567,086	22,864,722	3,297,636	21	17	38.12–38.12
	18	11,103,441	17,289,702	6,186,261	63	136	40.73–40.73
	20	34,667,432	52,106,931	17,439,499	210	111	38.06–44.66
British taurine	1	103,066,487	106,582,209	3,515,722	21	20	38.68–38.68
	3	30,373,903	33,782,646	3,408,743	21	79	40.79–40.79
	6	70,246,603	73,681,796	3,435,193	21	48	36.18–36.18
	8	1	4,480,553	4,480,552	42	34	38.87–39.89
	10	49,315,059	52,839,164	3,524,105	21	53	36.57–36.57
	15	3,488,628	9,262,742	5,774,114	63	15	37.34–40.79
	17	53,190,508	61,156,001	7,965,493	105	151	36.19–39.43
	21	1	5,473,762	5,473,761	63	83	36.04–40.93
	22	22,008,269	26,837,289	4,829,020	42	19	38.23–40.41
	28	35,161,062	38,282,522	3,121,460	21	29	36.55–36.55
Continental European taurine	1	16,598,522	24,306,959	7,708,437	84	31	45.10–46.26
	3	62,632,266	65,563,617	2,931,351	21	11	44.71–44.71
	9	61,967,748	68,119,665	6,151,917	63	54	44.71–50.91
	10	94,664,755	97,888,496	3,223,741	21	3	44.83–44.83
	14	30,880,542	38,346,797	7,466,255	63	79	43.5–46.90
	14	69,294,583	75,380,047	6,085,464	84	50	43.34–46.95
	20	21,145,977	23,172,452	2,026,475	21	26	49.44–49.44
	26	4,879,985	4,898,442	18,457	63	20	44.68–54.08

### 3.2 Gene and QTL Annotation

**FIGURE 4:**
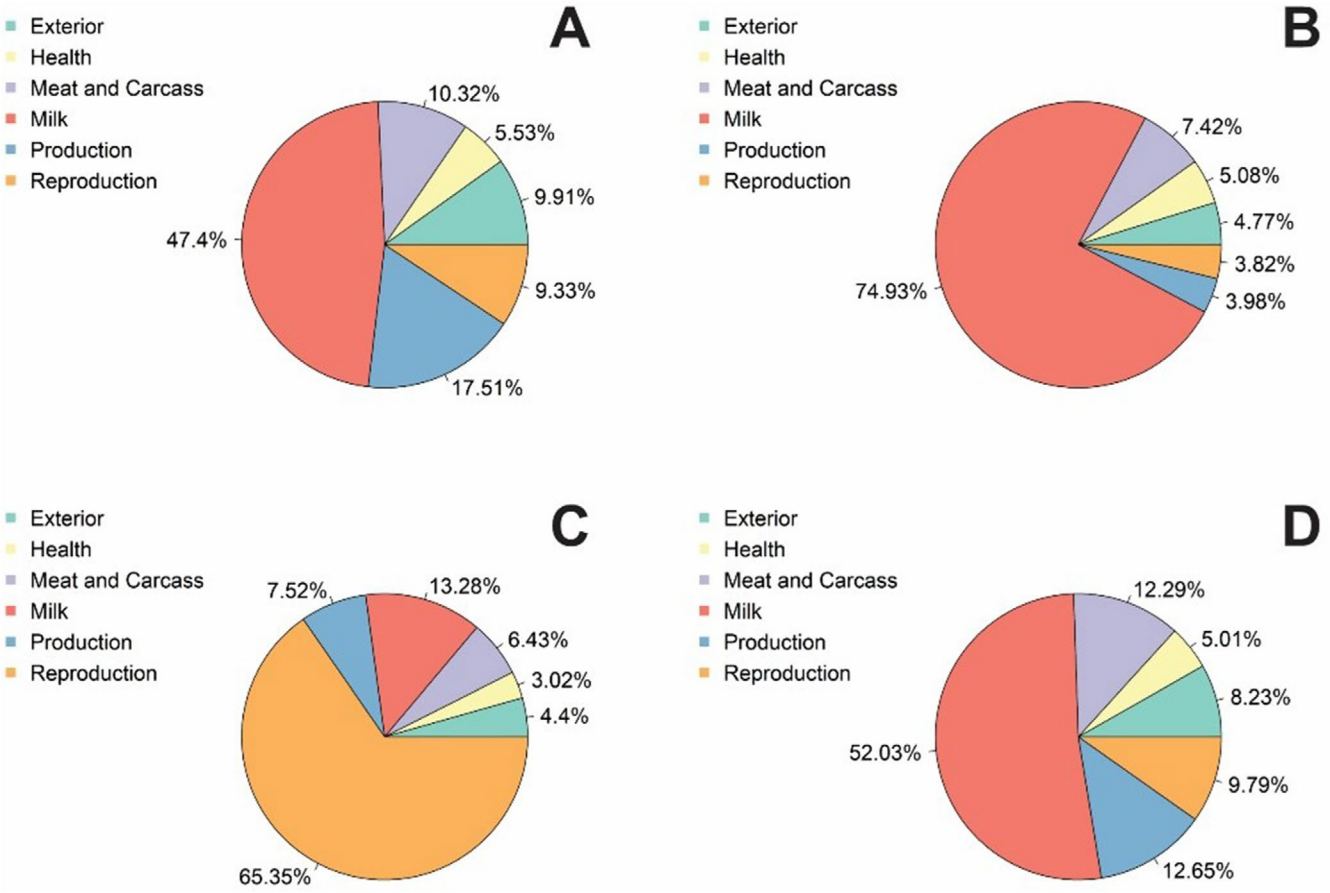
Distribution of QTL involvement across various physiological mechanisms. (A) Indicine, (B) Adapted taurine, (C) British taurine, (D) Continental European taurine across 3217 Montana Tropical animals. [Colour figure can be viewed at wileyonlinelibrary.com]

The annotation of genes and QTL in the top 1% regions of ancestry from the four biological types indicated milk production as the first biological mechanism followed by reproduction (Figure 4). Therefore, here we identified the genomic regions that demonstrate a deviance of average ancestry in the genome of the composite. For Indicine, the genes enriched are associated with milk‐related phenotypes, length of productive life and disease susceptibility. For tropical‐adapted taurine, the top enriched traits are milk composition, coat texture and carcass traits.

## 4 Discussion

This study provides significant insights into the population structure and genetic ancestry of the Montana Tropical composite breed, as well as the broader relationships among different cattle breeds. Through principal component analysis (PCA), ADMIXTURE analysis and local ancestry assessments, a detailed picture emerges of the genetic background influencing this composite population. In a previous study, Santos et al. (2024) have shown that selection pressure within the breed has been shaping the genomic clustering of the animals. Moreover, these authors identified a selective signature on BTAU 20 based on runs of homozygosity and integrated haplotype score (iHS).

PCA (Figure 1) revealed that Montana Tropical composite occupies an intermediate space between Indicine and Taurine ancestry, but closer to the Taurine cluster. This was expected since the breed association accepts a maximum of 37.5% of indicine ancestry on the pedigree for animal registration (https://montana.org.br). It is particularly intriguing that the African breeds Tuli and Senepol (both tropical adapted taurine breeds) clustered closely with the Montana Tropical group, suggesting similar genetic composition or convergent adaptive traits. Senepol is one of the main founder breeds of the tropical adapted taurine group. Tuli is a taurine breed reared in similar tropical conditions, which can show this convergent selection for adaptive traits.

The second principal component (PC2), which accounts for 4.81% of the variation, highlights differences within *Bos taurus* breeds, effectively distinguishing among Hereford, Angus and other Taurine breeds. This separation underscores the within diversity among *Bos taurus* breeds, influenced by demographic processes like genetic isolation, breed formation and breed‐specific selection (Upadhyay et al. 2017). Moreover, PC2 highlights the diversity within the Montana population, which can be related to clustering according to the number of generations inside the breed identified by Santos et al. (2024).

The ADMIXTURE results (Figure 2) corroborate the PCA findings, showing the heterogeneous ancestry composition of the Montana Tropical composites. At *K* = 2 and 4 levels, the relatively higher Taurine versus Indicine contributions align with the breed's selection goals for adaptability to harsh environments and productivity. The lack of dominance by any single ancestral breed underscores the composite's diverse genetic background, likely offering increased genetic adaptability. Generally, composite breeds offer genetic variation that is crucial in adaptation mechanisms (Barrett and Schluter 2008).

Detailed local ancestry analysis reveals an indicine ancestry average of 24% and a substantial contribution (62%) from three Taurine groups, indicating complementarity by incorporating desirable traits from different breeds. The total taurine ancestry consisted of 19.6% (±6.96) from tropical adapted taurine, 20.1% (±5.99) from British taurine and 21.9% (±7.79) from Continental European taurine. Comparing it to the expected breed composition based on pedigree (20.8% ± 8.5% Zebu, 50.5% ± 14.3% tropical adapted taurine, 21.1% ± 13.5% British taurine and 7.6% ± 5.1% of Continental European taurine) demonstrated a higher indicine contribution genomically than expected, probably related to environmental adaptation. Moreover, it seems that the methodology used was not able to correctly assign most of the adapted taurine counterpart, which probably is related to the inability to attribute 14% of the genome to any specific ancestry. As clustering approaches like ADMIXTURE capture common genetic ancestry rather than real causal contributions, the origin of adapted taurine ancestry in Montana could be trick due to some indicine contributions in SEN foundation, and also the ancestral Creole contribution to NEL and BRM (Utsunomiya et al. 2021).

Interestingly, specific autosomal regions, notably Autosome 6, contain a mix of Indicine, tropical‐adapted taurine and British taurine ancestry, demonstrating hotspots of genetic introgression that may confer advantageous traits for the composite's adaptability and production in harsh environments. Moreover, this architecture highlights how selection shapes the structure of the chromosome, taking advantage of the best genetic variations from each ancestor.

**FIGURE 5:**
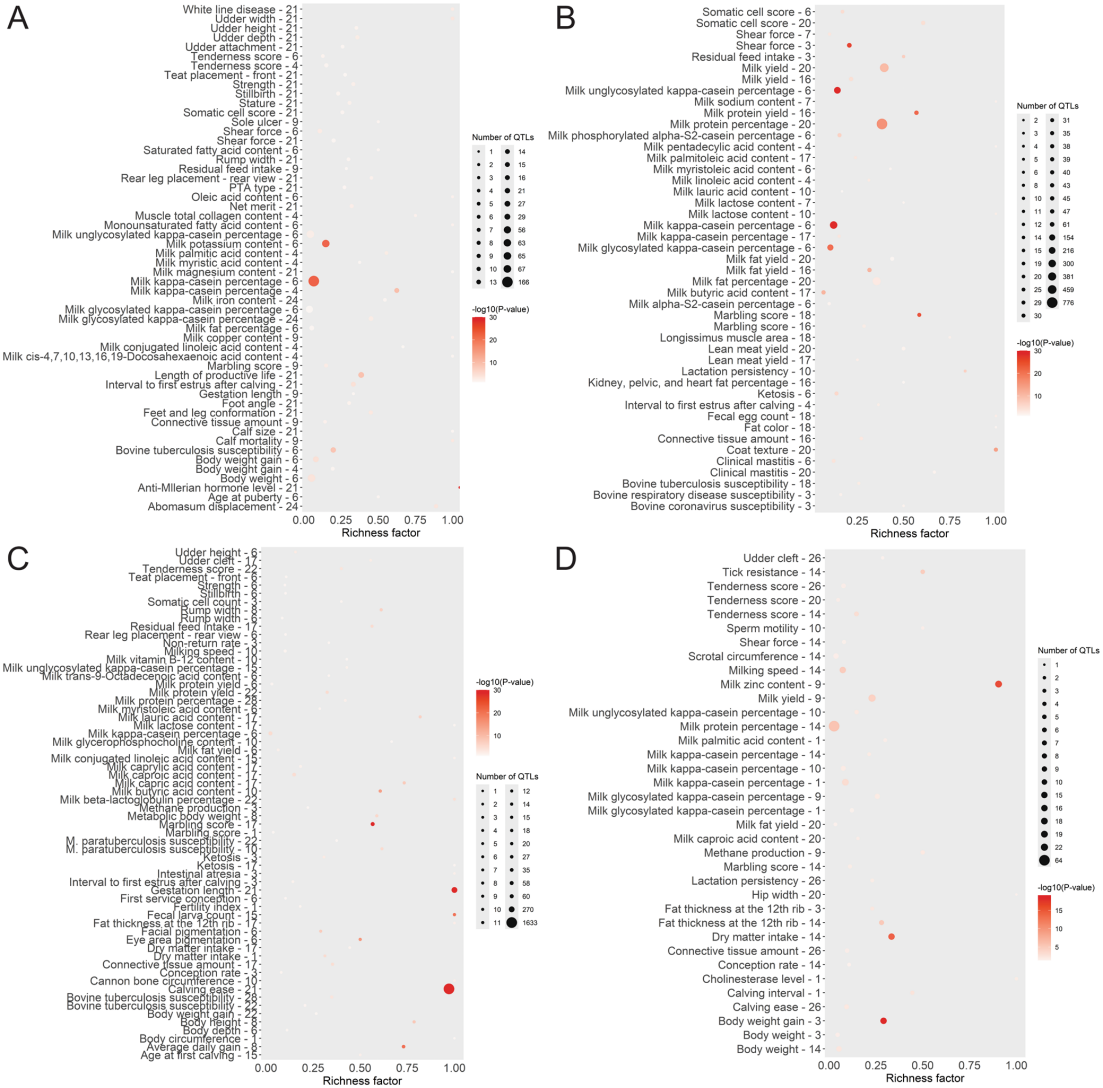
Bubble plot displaying the enrichment results for the enriched QTLs identified using the QTLs annotated within the candidate regions. (A) Indicine, (B) Adapted taurine, (C) British taurine, (D) Continental European taurine across 3217 Montana Tropical animals. [Colour figure can be viewed at wileyonlinelibrary.com]

Gene and QTL annotations within the top 1% ancestry regions (Table 2) indicate biological functions crucial for the production and survivability of the Montana Tropical composites. Enrichment analysis of the QTL regions showed Indicine contributions related to milk kappa casein percentage, length of productive life, Anti‐Mullerian hormone level and bovine tuberculosis susceptibility (Figure 5). Specifically, an Indicine ancestry region in 43.4 Mb on BTA4 is associated with carcass traits, as reported by Leal‐Gutiérrez et al. (2020). Additionally, a region of 39.5 Mb on BTA21 is associated with tick resistance. This is expected since Indicine are known for parasite resistance (Seifert 1971).

The top 1% adapted taurine ancestry regions included a region on BTA20 associated with coat texture and already identified as a selection signature in this population (Santos et al. 2024). A region in 5.7 Mb on BTA3 is associated with meat tenderness (Leal‐Gutiérrez et al. 2020). Additionally, a region in 12.8 Mb on BTA4 is associated with milk protein yield and percentage (Pedrosa et al. 2021). The enrichment analysis (Figure 5) showed the highest richness factor for coat texture, followed by marbling score and the lowest *p*‐values for shear force and milk composition traits.

For the British taurine ancestry regions, they consisted of a region in 103 Mb on BTA1 associated with intermuscular fat content (Leal‐Gutiérrez et al. 2020). Additionally, a region in 30.3 Mb on BTA3 was reported to be associated with calving interval (Rios et al. 2023). The QTL enrichment analysis (Figure 5) highlighted calving ease, gestation length, fertility index, average daily gain and marbling score with high richness factors and very low *p*‐values.

Finally, the ancestry regions originating from Continental European taurine consisted of regions associated with several phenotypes. A region in 16.5 Mb on BTA1 is associated with marbling and meat tenderness (Leal‐Gutiérrez et al. 2020). Additionally, a region in 37.9 Mb on BTA14 is associated with milk protein content (Pedrosa et al. 2021). QTL enrichment analysis highlights milk zinc content, dry matter intake and body weight gain for Continental European taurine contributions.

Mulim et al. (2022) characterised another Brazilian composite beef cattle breed known as Purunã, which was formed by crossing Angus, Charolais, Canchim and Caracu. These authors identified heterozygote islands harbouring genes involved in growth pathways, carcass weight, meat and carcass quality and marbling deposition. This four‐breed composite population had low consistency of gametic phases with the founder breeds; therefore, multi‐breed genomic evaluation is likely not feasible (Mulim et al. 2022). Composite breeds formed by more than two breeds and with multiple taurine and indicine founders can have a different genetic architecture than previously studied two‐breed composite populations (Grigoletto et al. 2020). Here, we confirm how complex it is to understand the genetic composition of a composite population from multiple breeds such as Montana Tropical.

These findings of ancestry regions enrichment underscore the practical benefits of maintaining such genetic diversity in composite breeds, targeting economic traits essential for sustainability in diverse production environments. Moreover, these results highlight as a crossbreeding coupled with a well‐designed animal breeding programme can shape the genetic architecture of the population to better explore the genetic variability available. Here, we see from a genomic perspective how crossbreeding and composite breed selection can conserve important genetic variation for animal production in specific raising conditions.

## 5 Conclusions

This study unravels the complex genetic ancestry of the Montana Tropical composite, highlighting the effective blend of ancestral gene pools that enhance key production and adaptation traits. These results can inform targeted breeding programmes and conservation strategies, ensuring the optimisation of such composite breeds for future agricultural challenges.

## Conflicts of Interest

The authors declare no conflicts of interest.

## Supporting information


**Figure S1:** Cross‐validation error of ADMIXTURE analyses across *K* values from 1 to 19.

## Data Availability

The data that support the findings of this study are available on request from the corresponding author. The data are not publicly available due to privacy or ethical restrictions.
