# Supplementary figures and images for: Genomic Analysis of Taurine and Indicine Ancestry in the Montana Tropical Composite Population
Source: J Anim Breed Genet. 2025 Sep 29;143(2):273–82. doi: 10.1111/jbg.70017 (PMC12887133; doi:10.1111/jbg.70017)

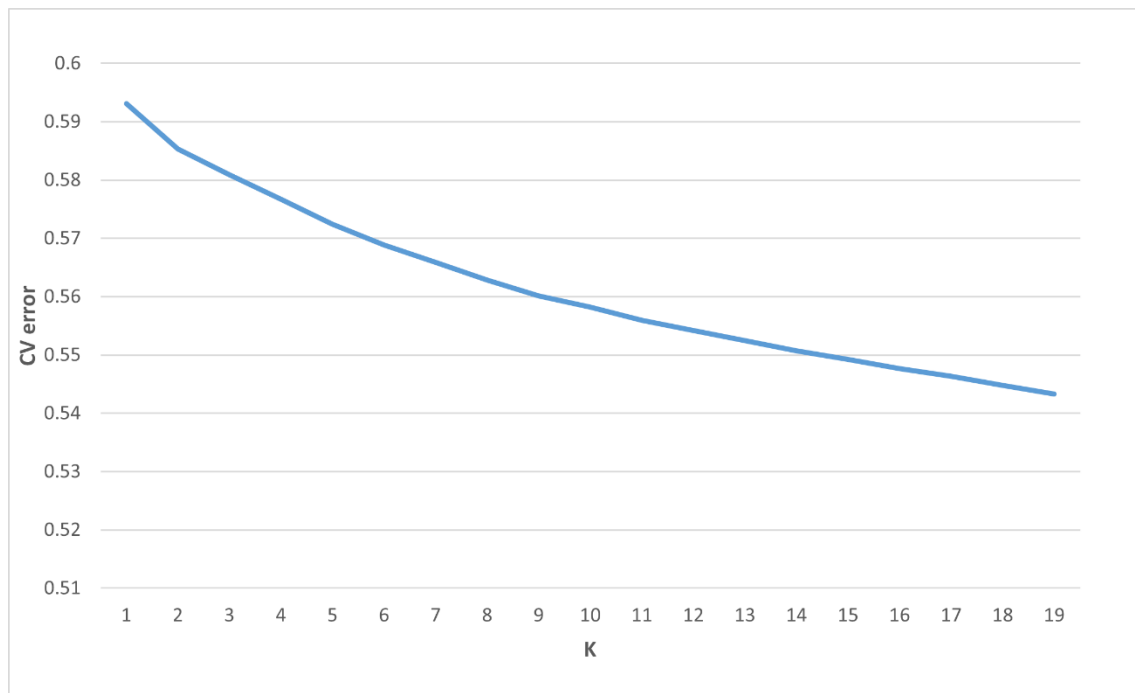

**Supplementary figure 1.** Cross-validation error of ADMIXTURE analyses across K values from 1 to 19 .

Supplement: Supplementary file 1 — Figure S1: Cross‐validation error of ADMIXTURE analyses across K values from 1 to 19. [file JBG-143-273-s001.pdf]
